# State-Level Prevalence of Cigarette Smoking and Treatment Advice, by Disability Status, United States, 2004

**Published:** 2007-09-15

**Authors:** Brian S Armour, Vincent A Campbell, John E Crews, Roland A Richard, Ann Malarcher, Emmanuel Maurice

**Affiliations:** Division of Health and Human Development, National Center on Birth Defects and Developmental Disabilities, Centers for Disease Control and Prevention; Division of Health and Human Development, National Center on Birth Defects and Developmental Disabilities, Centers for Disease Control and Prevention, Atlanta, Georgia; Division of Health and Human Development, National Center on Birth Defects and Developmental Disabilities, Centers for Disease Control and Prevention, Atlanta, Georgia; Division of Health and Human Development, National Center on Birth Defects and Developmental Disabilities, Centers for Disease Control and Prevention, Atlanta, Georgia; Office on Smoking and Health, National Center for Chronic Disease Prevention and Health Promotion, Centers for Disease Control and Prevention, Atlanta, Georgia; Office on Smoking and Health, National Center for Chronic Disease Prevention and Health Promotion, Centers for Disease Control and Prevention, Atlanta, Georgia

## Abstract

**Introduction:**

To our knowledge, no study has determined whether smoking prevalence is higher among people with disabilities than among people without disabilities across all U.S. states. Neither do we know whether people with disabilities and people without disabilities receive the same quality of advice about tobacco-cessation treatment from medical providers.

**Methods:**

We analyzed data from the 2004 Behavioral Risk Factor Surveillance System to estimate differences between people with and people without disabilities in smoking prevalence and the receipt of tobacco-cessation treatment advice from medical providers.

**Results:**

We found that smoking prevalence for people with disabilities was approximately 50% higher than for people without disabilities. Smokers with disabilities were more likely than smokers without disabilities to have visited a medical provider at least once in the previous 12 months and to have received medical advice to quit. More than 40% of smokers with disabilities who were advised to quit, however, reported not being told about the types of tobacco-cessation treatment available.

**Conclusion:**

Ensuring that people with disabilities are included in state-based smoking cessation programs gives states an opportunity to eliminate health disparities and to improve the health and wellness of this group. Ways to reduce unmet preventive health care needs of people with disabilities include provider adoption of the Public Health Service's *clinical practice guideline* for treating tobacco use and dependence and the provision of smoking cessation services that include counseling and effective pharmaceutical treatment.

## Introduction

Disability affects more than 50 million Americans, and annual health care expenditures and productivity losses for people with disabilities exceed $300 billion (1). A compounding factor in this public health issue is that smoking prevalence is higher among people with disabilities than among people without disabilities, as was found in one population-based study of adults in Massachusetts (2). Other research indicates that the prevalence of cigarette smoking is higher in some socioeconomic and demographic groups than in others and that not all populations, including people with disabilities, receive the same level of preventive health care (1,3). To our knowledge, no study has determined whether the findings in the Massachusetts study extend to other states. From a policy standpoint, identifying disparities in smoking prevalence is important to determining how best to direct resources to reduce these disparities. Moreover, reducing health disparities is especially relevant, given the recent publication of *the 2005 Surgeon General's Call to Action To Improve the Health and Wellness of People with Disabilities* (4).

Health care costs for people with disabilities and for the elderly account for a large proportion of Medicare and Medicaid expenditures (5). In 2001, approximately 40% of Medicare expenditures were for inpatient hospital care for these groups (6). Expenses related to the chronic conditions of 5% of Medicare fee-for-service beneficiaries accounted for 34% of the expense for all beneficiaries. Although people who have a disability or are elderly make up one-quarter of Medicaid's recipients, they accounted for approximately 70% of the program's expenditures in 2004 (7). Long-term care for people whose disability prevents them from living independently accounted for approximately one-third of Medicaid spending in 2004.

According to the Congressional Budget Office, Medicare and Medicaid expenditures may grow at unsustainable rates (6). Chronic disease management has been suggested as a means of containing the growth rate in Medicare costs, and the National Governors Association recommends that states consider health promotion activities to prevent chronic disease as a means of controlling Medicaid expenditures (8).

Smoking, which harms nearly every organ of the body and causes many cancers, cardiovascular diseases, and respiratory illnesses, is the leading preventable cause of morbidity and mortality and results in approximately 440,000 deaths annually in the United States (9,10). *Healthy People 2010* (1) recommends that states target tobacco-related illnesses through tobacco-control programs to reduce disease, disability, and death related to tobacco use by preventing the initiation of tobacco use, promoting cessation, eliminating exposure to secondhand smoke, and identifying and working to eliminate disparities in tobacco use among different populations. The *Healthy People 2010* objectives are identical to several of the National Governors Association recommendations designed to reduce tobacco use.

The purpose of our study was to identify disparities in current smoking prevalence among people with and people without disabilities and to estimate the prevalence and assess the quality of advice on tobacco-cessation treatment given by medical providers to this population by state.

## Methods

We used data from the 2004 Behavioral Risk Factor Surveillance System (BRFSS) from the 50 U.S. states; the District of Columbia; and the two U.S. territories for which data were available, Puerto Rico and the U.S. Virgin Islands. The BRFSS is a state-based, random-digit–dialed, telephone survey of the U.S. civilian noninstitutionalized population aged 18 years or older (11,12). Conducted by all states with assistance from the Centers for Disease Control and Prevention (CDC), the survey is completed by trained interviewers who collect comprehensive data on demographics, health, behavioral health risks, and disease prevention behaviors. The data are used to quantify state-level prevalences of major behavioral health risks associated with premature morbidity and mortality and to evaluate their impact. In turn, this information is used in the development of state health promotion and disease prevention programs. A detailed description of the compilation and use of BRFSS data is available at www.cdc.gov/brfss/.

### Definitions 

**Disability**

The 2004 BRFSS questionnaire included two questions on disability screening: "Are you limited in any way in any activities because of physical, mental, or emotional problems?" and "Do you now have any health problem that requires you to use special equipment, such as a cane, a wheelchair, a special bed, or a special telephone?" We defined respondents as having a disability if they answered yes to either of these questions. Data on people who did not respond, refused to respond, or whose responses were missing were excluded from the analysis.

**Current smoking**

The survey used two questions to determine cigarette use: "Have you smoked at least 100 cigarettes in your entire life?" and "Do you now smoke cigarettes every day, some days, or not at all?" We defined current smokers as people who reported smoking at least 100 cigarettes during their lifetime and who currently smoke every day or some days. Data on people who did not respond, refused to respond, or whose responses were missing were excluded from the analysis.

**Smoking cessation treatment advice**

Each year, states may choose to incorporate optional modules of questions on specific topics into their BRFSS survey. In 2004, 20 states and the U.S. Virgin Islands included questions from the tobacco-cessation module. Respondents were asked, "In the past 12 months, how many times have you seen a doctor, nurse, or other health professional to get any kind of care for yourself?" Respondents who had seen a doctor were also asked, "In the past 12 months, on how many visits were you advised to quit smoking by a doctor or other health care provider?" We defined people as having received advice to quit smoking if they reported having received such advice on at least one visit. Data on people who did not respond, refused to respond, or whose responses were missing were excluded from the analysis.

Respondents who had seen a health care provider in the previous 12 months were also asked, "On how many visits did your doctor, nurse, or other health professional recommend or discuss medication to assist you with quitting smoking, such as nicotine gum, patch, nasal spray, inhaler, lozenge, or prescription medication such as Wellbutrin/Zyban/Bupropion?" and "On how many visits did your doctor or health provider recommend or discuss methods and strategies other than medication to assist you with quitting smoking?" We defined respondents as having been counseled on smoking cessation treatment options if they reported receiving this information on at least one visit. Data on people who did not respond, refused to respond, or whose responses were missing were excluded from the analysis.

### Analysis

We used a SAS-callable version of SUDAAN (Research Triangle Institute, Research Triangle Park, North Carolina) to obtain state-level prevalences of disability, current smoking, and advice given by a medical provider on smoking cessation and on medication to assist in quitting. For estimates of standard errors and 95% confidence intervals (CIs), the data were weighted to account for differential probability of selection and, in part, to adjust for nonresponse. Estimates were age-adjusted to the 2000 U.S. standard population (13,14). Estimates were not reported if the number of responses to questions on smoking status, smoking cessation advice, and treatment options were less than 50 or the related CI half-width was greater than 10. Hawaii was not included in our analysis, because data were not available for 2004.

## Results

In 2004, the median age-adjusted prevalence of current cigarette smoking in the 49 states for which data were available and in the District of Columbia, Puerto Rico, and the U.S. Virgin Islands was higher for people with disabilities (median = 29.9%) than for people without disabilities (median = 19.8%) (Table 1). Smoking prevalence for people with disabilities was highest in Delaware (39.4%; 95% CI, 33.4–45.4) and lowest in Puerto Rico (16.5%; 95% CI, 11.9–21.1). In a comparison of smoking among people with and without disabilities, Delaware had the highest difference in smoking prevalence (17.1 percentage points), and Puerto Rico had the lowest (4.3 percentage points).

For the 20 states that administered the smoking cessation module in 2004, we calculated both age-adjusted and unadjusted estimates (Table 2). Although we present both sets of data in the table for comparison, we discuss only the age-adjusted estimates. According to these estimates, smokers with disabilities were more likely (median = 87.2%) than smokers without disabilities (median = 75.5%) to report having visited a health care provider at least once in the previous 12 months. Of this group, smokers with disabilities were more likely (median = 70.7%) than smokers without disabilities (median = 66.9%) to have been advised by their provider about smoking cessation. And of smokers receiving such advice, those with disabilities were more likely (59.6%) than those without disabilities (51.0%) to have discussed medications and other methods to quit smoking with their providers.

## Discussion

For the first time since the inception of national health objectives*, Healthy People 2010 *includes a goal specifically for people with disabilities (1). That goal is to promote the health of people with disabilities, prevent secondary conditions, and reduce health disparities. The chapter on tobacco use in *Healthy People 2010* sets the year 2010 target for cigarette smoking prevalence at 12% — a 50% reduction in prevalence — and points out that current levels of cigarette use are highest among men and certain racial and ethnic populations, including American Indians and Alaskan Natives (1). Our findings reveal a substantially higher prevalence of cigarette smoking among people with disabilities than among people without disabilities, indicating that people with disabilities must be included with other minority populations as a target group in tobacco-cessation activities if states hope to meet the *Healthy People 2010* smoking prevalence target.

Comprehensive tobacco control, including health insurance coverage for cessation counseling and pharmaceutical treatments, is effective in preventing and reducing tobacco use (15-18). When health care providers must address multiple acute health problems, however, their time spent on discussing preventive treatment options with patients may be less than optimal, which may lead to reduced quality of care for people with disabilities (19). The underuse of preventive health care for people with disabilities may also be a result of patient-provider miscommunication, lack of patient adherence, patient access problems (e.g., transportation), lack of provider training, office staff's lack of knowledge on how to accommodate people with disabilities, poor specialist care coordination, and insufficient financial incentives (19-31).

People with disabilities are less likely than people without disabilities to receive preventive health care and so are more susceptible to illness and disease (19-31). The result may be a decline in health, which can lead to reduced levels of activity and increased functional dependence. The earlier study in Massachusetts (2) suggests that smokers with disabilities are more likely than smokers without disabilities to receive medical advice to quit. Information is not available, however, on whether the quality of advice given to these two groups is comparable. Although our findings concur with the Massachusetts study, they also reveal that more than 40% of smokers with disabilities who were advised to quit did not receive information about medication and other tobacco-cessation treatments.

To improve the health of people with disabilities, the 50 states, the District of Columbia, and the U.S. territories should provide coverage under Medicaid for all recommended tobacco-dependence treatments (32). Aside from the advantages to public health, reducing smoking prevalence among people with disabilities is fiscally prudent. Disability is associated with poverty (33), and smoking rates of participants in state-funded programs, such as Medicaid, that serve these people are 50% higher than those for the U.S. adult population (32,34). Medicaid expenditures attributable to smoking exceed $12 billion annually (35), a cost that could be cut if states invested in smoking cessation efforts that reduced the prevalence of smoking (36).

State revenues from tobacco settlement money and tobacco excise taxes totaled $21.2 billion in fiscal year 2006 (37). Despite this income stream and reported increases in state smoking cessation expenditures associated with reductions in smoking prevalence (36), the amount of money that states spent on tobacco control in 2006 totaled only $551 million, approximately one-third of the minimum amount recommended by CDC to prevent and reduce tobacco use and minimize health-related harm and cost (37). For fiscal year 2007, only three states — Colorado, Delaware, and Maine — achieved CDC's recommended minimum per capita investment for tobacco-control programs (38). To effectively prevent and reduce tobacco use among people with disabilities, states will need to promote health system changes that reduce barriers limiting the ability of these people to access and use preventive health care. Efforts and funding to reduce disparities in smoking prevalence among people with disabilities must be increased if more states are to achieve the *Healthy People 2010* goal of reducing smoking prevalence to 12% or less.

To our knowledge, our study is the first to quantify state-level differences in smoking prevalence among people with and without disabilities and to identify opportunities to improve the quality of tobacco-cessation counseling given to people with disabilities. Despite this contribution, our study has several limitations. First, the BRFSS survey may understate the true prevalence of disability, because it excludes the institutionalized population, people in households without telephones, and people whose disability prevents them from answering the telephone. Second, BRFSS data are based on self-reports and have not been validated. Third, BRFSS questions used to define disability do not indicate type or severity. Without this information, our results may be conservative, given that certain types (e.g., psychiatric conditions) and levels of severity of disability are associated with the increased likelihood of smoking and with greater nicotine dependence (39-42).

Fourth, other factors, including sociodemographic characteristics such as poverty, might be associated with smoking (36). Although the BRFSS does not identify respondents who live in poverty, it does report income ranges. In 2004, 5.3% of BRFSS respondents reported income below $10,000. Using this income threshold as a proxy for poverty, we found that smoking prevalence was 14.9 percentage points higher for impoverished respondents with disabilities than for impoverished respondents without disabilities (36.7% vs 22.6%; *P* < .01). Smoking prevalence was 4.3 percentage points higher for respondents with disabilities with annual incomes of $10,000 or more than their counterparts with this level of income (24.4% vs 20.1%; *P* < .01). When we adjusted these estimates for age, the disparities were larger. We also reexamined, by poverty status, the state-level differences in the frequency and quality of tobacco-cessation treatment advice given by health care providers to people with and without disabilities. We found that impoverished smokers with disabilities were more likely than impoverished smokers without disabilities to receive medical advice to quit and that providers were more likely to discuss medication and other tobacco-cessation treatments with these patients. Accounting for the potential confounding effects of other factors associated with smoking using multivariate analytical techniques is a direction for future work.

Finally, characterizing the causality between smoking and disability was beyond the scope of this analysis. This information would be valuable because smoking rates are higher for people with disabilities than for people without disabilities, and people with disabilities who smoke increase their risk of developing chronic conditions that might adversely interact with their primary disabling condition.

In the belief that reducing rates of debilitating chronic diseases may slow the growth rate in health care expenditures, interest has focused on reducing disparities in the quality of preventive health care. This work has centered on identifying and eliminating racial and ethnic differences, but people with disabilities are another vulnerable population whose preventive health care needs often go unmet. Disparities in smoking prevalence and the use of preventive medical services put people, particularly those with disabilities, at risk for declining health, decreased levels of activity, and increased functional dependence. Health promotion activities to prevent or reduce the onset of chronic diseases among people with disabilities may slow the growth rate in health care costs by reducing hospital admissions and delaying or preventing nursing home entry.

Ensuring that people with disabilities are included in state-based smoking cessation programs gives states an opportunity to eliminate disparities and improve the quality of preventive health care for people with disabilities. This action would meet the *Healthy People 2010* objectives on disability and tobacco use and the recommendations of the National Governors Association (7) and could reduce the growth rate in health care expenditures. Ways to reduce unmet preventive health care needs of people with disabilities include provider adoption of the Public Health Service's clinical practice guideline for treating tobacco use and dependence and the provision of smoking cessation services that include counseling and effective pharmaceutical treatment. Given the barriers that limit access to and use of preventive health care services, the 1-800-QUITNOW National Network of Tobacco Cessation Quitlines and the www.smokefree.gov Web site may be particularly important in assisting people with disabilities to quit smoking.

The inclusion of people with disabilities in smoking cessation programs will require overcoming the many barriers to preventive care that they experience. For most states, this action will require reversing recent trends and following the examples set by Colorado, Delaware, and Maine by funding at least the minimum per capita amount that CDC recommends for tobacco-control programs. In the absence of meeting the CDC recommendation, the preventive health care needs of these people will continue to go unmet.

## Figures and Tables

**Table 1:** Smoking Prevalence Among Adults^a^, by Disability Status, Behavioral Risk Factor Surveillance System, 49 States^b^, District of Columbia, Puerto Rico, and the U.S. Virgin Islands^c^, 2004

State, District, Territory	With Disability		Without Disability	

	Unadjusted% (95% CI)	Age-Adjusted% (95% CI)	Unadjusted% (95% CI)	Age-Adjusted% (95% CI)
Alabama	29.7 (26.1-33.3)	34.3 (29.5-39.1)	23.7 (21.7-25.7)	23.3 (21.3-25.3)
Alaska	29.7 (23.9-35.5)	31.9 (25.4-38.4)	23.3 (20.7-25.9)	21.7 (19.2-24.2)
Arizona	25.7 (19.4-32.0)	30.2 (21.9-38.5)	17.1 (14.8-19.4)	16.8 (14.6-19.0)
Arkansas	27.3 (24.0-30.6)	32.1 (27.5-36.7)	24.6 (22.7-26.5)	24.3 (22.5-26.1)
California	18.2 (15.0-21.4)	22.5 (18.3-26.7)	14.1 (12.7-15.5)	13.6 (12.2-15.0)
Colorado	23.8 (20.0-27.6)	28.3 (23.2-33.4)	19.5 (17.8-21.2)	18.6 (17.0-20.2)
Connecticut	22.4 (19.3-25.5)	27.9 (23.6-32.2)	17.1 (15.7-18.5)	17.0 (15.6-18.4)
Delaware	33.0 (28.2-37.8)	39.4 (33.4-45.4)	22.9 (20.8-25.0)	22.3 (20.3-24.3)
Florida	23.9 (20.6-27.2)	30.8 (26.3-35.3)	19.2 (17.5-20.9)	19.6 (17.9-21.3)
Georgia	23.2 (19.8-26.6)	26.4 (22.0-30.8)	19.3 (17.5-21.1)	18.6 (16.9-20.3)
Idaho	23.8 (20.8-26.8)	28.5 (24.5-32.5)	16.0 (14.6-17.4)	15.5 (14.1-16.9)
Illinois	23.5 (19.7-27.3)	28.6 (23.7-33.5)	21.9 (20.1-23.7)	21.3 (19.6-23.0)
Indiana	29.3 (26.2-32.4)	35.4 (31.6-39.2)	23.9 (22.5-25.3)	23.3 (22.0-24.6)
Iowa	22.3 (19.1-25.5)	29.5 (24.4-34.6)	20.7 (19.2-22.2)	20.5 (19.0-22.0)
Kansas	23.9 (21.6-26.2)	29.0 (25.7-32.3)	19.1 (17.9-20.3)	18.6 (17.5-19.7)
Kentucky	31.5 (28.4-34.6)	35.1 (31.1-39.1)	26.7 (24.5-28.9)	26.0 (23.9-28.1)
Louisiana	29.4 (26.5-32.3)	33.6 (30.0-37.2)	22.2 (20.9-23.5)	21.6 (20.4-22.8)
Maine	25.8 (21.7-29.9)	30.2 (24.9-35.5)	19.7 (17.8-21.6)	19.9 (18.0-21.8)
Maryland	24.9 (20.3-29.5)	28.6 (22.7-34.5)	18.2 (16.4-20.0)	17.7 (16.0-19.4)
Massachusetts	24.0 (21.2-26.8)	28.2 (24.5-31.9)	17.5 (16.1-18.9)	17.2 (15.9-18.5)
Michigan	27.4 (24.1-30.7)	32.6 (28.3-36.9)	22.2 (20.5-23.9)	21.6 (20.0-23.2)
Minnesota	21.3 (18.5-24.1)	24.9 (21.3-28.5)	20.5 (18.8-22.2)	19.7 (18.1-21.3)
Mississippi	28.7 (25.8-31.6)	33.3 (29.5-37.1)	23.1 (21.4-24.8)	22.4 (20.8-24.0)
Missouri	25.5 (22.3-28.7)	29.9 (25.5-34.3)	23.7 (21.7-25.7)	23.0 (21.1-24.9)
Montana	23.7 (20.5-26.9)	26.6 (22.3-30.9)	19.1 (17.4-20.8)	18.9 (17.2-20.6)
Nebraska	24.0 (21.2-26.8)	29.4 (25.5-33.3)	19.5 (18.2-20.8)	19.2 (17.9-20.5)
Nevada	28.3 (23.1-33.5)	32.7 (26.2-39.2)	22.3 (19.8-24.8)	22.0 (19.6-24.4)
New Hampshire	28.6 (25.2-32.0)	33.1 (28.8-37.4)	20.0 (18.4-21.6)	19.6 (18.1-21.1)
New Jersey	23.2 (20.9-25.5)	27.5 (24.3-30.7)	18.3 (17.3-19.3)	18.1 (17.1-19.1)
New Mexico	24.4 (21.5-27.3)	28.6 (24.7-32.5)	19.1 (17.7-20.5)	18.7 (17.3-20.1)
New York	25.1 (22.0-28.2)	28.9 (25.0-32.8)	18.8 (17.4-20.2)	18.5 (17.1-19.9)
North Carolina	26.4 (24.4-28.4)	30.8 (28.1-33.5)	22.5 (21.4-23.6)	21.8 (20.8-22.8)
North Dakota	18.9 (15.2-22.6)	27.8 (21.9-33.7)	20.1 (18.2-22.0)	19.9 (18.0-21.8)
Ohio	31.5 (26.5-36.5)	38.0 (32.0-44.0)	24.7 (22.2-27.2)	24.4 (22.0-26.8)
Oklahoma	29.2 (26.6-31.8)	34.8 (31.3-38.3)	25.3 (23.8-26.8)	24.7 (23.2-26.2)
Oregon	25.5 (22.5-28.5)	30.0 (26.2-33.8)	18.3 (16.8-19.8)	17.9 (16.4-19.4)
Pennsylvania	27.0 (24.1-29.9)	32.6 (28.7-36.5)	21.9 (20.4-23.4)	22.0 (20.5-23.5)
Rhode Island	23.5 (20.0-27.0)	27.7 (22.8-32.6)	21.1 (19.1-23.1)	20.8 (18.9-22.7)
South Carolina	29.1 (26.1-32.1)	34.9 (31.0-38.8)	23.3 (21.8-24.8)	22.6 (21.2-24.0)
South Dakota	25.6 (22.3-28.9)	31.3 (26.3-36.3)	19.4 (18.0-20.8)	19.2 (17.8-20.6)
Tennessee	31.6 (27.5-35.7)	34.5 (29.1-39.9)	24.7 (22.5-26.9)	24.1 (22.0-26.2)
Texas	26.7 (23.4-30.0)	30.4 (26.3-34.5)	19.3 (17.9-20.7)	18.5 (17.1-19.9)
Utah	15.0 (12.2-17.8)	17.3 (13.9-20.7)	9.5 (8.4-10.6)	9.0 (8.0-10.0)
Vermont	24.6 (22.0-27.2)	29.4 (26.0-32.8)	18.8 (17.5-20.1)	18.5 (17.2-19.8)
Virginia	25.0 (21.5-28.5)	30.3 (25.5-35.1)	20.2 (18.5-21.9)	19.4 (17.8-21.0)
Washington	24.6 (23.0-26.2)	28.5 (26.4-30.6)	17.6 (16.7-18.5)	16.9 (16.1-17.7)
West Virginia	29.7 (26.4-33.0)	37.8 (33.3-42.3)	25.7 (23.6-27.8)	25.5 (23.4-27.6)
Wisconsin	22.1 (18.6-25.6)	26.6 (22.1-31.1)	21.9 (20.2-23.6)	21.4 (19.7-23.1)
Wyoming	25.7 (22.3-29.1)	29.6 (25.1-34.1)	21.0 (19.3-22.7)	20.6 (19.0-22.2)
Puerto Rico	12.3 (9.2-15.4)	16.5 (11.9-21.1)	12.7 (11.1-14.3)	12.2 (10.7-13.7)
District of Columbia	23.3 (18.0-28.6)	26.9 (20.7-33.1)	20.5 (18.3-22.7)	20.0 (17.9-22.1)
U.S. Virgin Islands	NR	NR	9.0 (7.5-10.5)	8.6 (7.2-10.0)
Median (49 States, DC, Territories)	25.1	29.9	20.2	19.8

CI indicates confidence interval; NR, not reported.

a People aged ≥18 years.

b Hawaii completed 3 of 12 months of interviews in 2004; these data are not available in the aggregate 2004 data set.

c BRFSS does not report data if the sample size is fewer than 50 or CI is greater than 10. Consequently, prevalence estimates for some territories are not reported.

**Table 2:** Age-Adjusted and Unadjusted Prevalence of Smoking Cessation Advice Among Adult^a^ Current Smokers, by Disability Status, Behavioral Risk Factor Surveillance System, 20 States^b^, 2004

State	Age-Adjusted Prevalence Estimates										

	With Disability					Without Disability					

	Had a Medical Encounter % (95% CI)	Received Quit Advice % (95% CI)		Discussed Tx Options % (95% CI)		Had a Medical Encounter % (95% CI)		Received Quit Advice % (95% CI)		Discussed Tx Options % (95% CI)	
Arizona	93.5 (89.2-97.8)	NR		NR		78.6 (72.4-84.8)		56.8 (48.8-64.8)		45.5 (36.1-54.9)	
Arkansas	86.2 (79.2-93.2)	62.2 (53.8-70.6)		NR		78.5 (74.9-82.1)		58.2 (53.1-63.3)		50.5 (43.7-57.3)	
Colorado	86.2 (79.3-93.1)	82.5 (74.9-90.1)		NR		69.8 (65.2-74.4)		69.6 (64.3-74.9)		54.2 (47.3-61.1)	
Delaware	92.7 (88.4-97.0)	66.8 (56.9-76.7)		NR		84.0 (80.1-87.9)		72.8 (67.8-77.8)		54.4 (47.7-61.1)	
Iowa	88.1 (81.2-95.0)	69.9 (60.4-79.4)		NR		75.4 (71.7-79.1)		58.7 (54.0-63.4)		50.9 (45.1-56.7)	
Kentucky	87.0 (82.7-91.3)	71.9 (65.2-78.6)		57.8 (49.5-66.1)		66.5 (61.9-71.1)		68.5 (63.1-73.9)		56.3 (49.4-63.2)	
Louisiana	80.0 (74.8-85.2)	67.0 (58.7-75.3)		53.0 (44.0-62.0)		68.7 (65.7-71.7)		61.6 (57.7-65.5)		50.3 (44.8-55.8)	
Montana	88.1 (82.2-94.0)	74.5 (67.8-81.2)		NR		76.1 (71.7-80.5)		63.6 (57.8-69.4)		48.2 (40.8-55.6)	
Nebraska	85.2 (79.7-90.7)	68.0 (60.3-75.7)		62.9 (53.2-72.6)		73.7 (70.4-77.0)		60.0 (55.7-64.3)		46.3 (40.5-52.1)	
New Jersey	89.4 (85.2-93.6)	75.0 (69.3-80.7)		55.3 (46.8-63.8)		77.4 (74.6-80.2)		68.7 (65.4-72.0)		49.4 (45.4-53.4)	
New York	84.4 (78.2-90.6)	72.8 (65.0-80.6)		62.6 (53.9-71.3)		72.8 (68.9-76.7)		69.3 (64.9-73.7)		53.5 (47.9-59.1)	
North Carolina	89.3 (86.3-92.3)	71.7 (66.7-76.7)		60.5 (54.1-66.9)		73.6 (71.2-76.0)		70.5 (67.7-73.3)		59.7 (56.2-63.2)	
North Dakota	86.9 (78.9-94.9)	NR		NR		71.5 (66.5-76.5)		59.6 (53.3-65.9)		49.9 (41.6-58.2)	
Ohio	85.2 (76.0-94.4)	68.9 (59.1-78.7)		NR		79.6 (74.6-84.6)		67.1 (60.4-73.8)		45.7 (37.5-53.9)	
South Carolina	87.3 (81.4-93.2)	71.4 (65.2-77.6)		52.9 (44.4-61.4)		76.6 (73.4-79.8)		67.3 (63.4-71.2)		51.1 (46.0-56.2)	
Texas	85.8 (80.6-91.0)	68.7 (60.9-76.5)		NR		66.0 (61.8-70.2)		58.8 (53.8-63.8)		42.1 (35.7-48.5)	
Virginia	92.3 (88.0-96.6)	70.1 (61.7-78.5)		64.9 (55.6-74.2)		78.3 (74.3-82.3)		66.7 (61.5-71.9)		56.4 (49.4-63.4)	
West Virginia	92.4 (89.1-95.7)	74.9 (68.5-81.3)		59.6 (50.8-68.4)		77.9 (73.6-82.2)		68.3 (63.1-73.5)		55.8 (49.2-62.4)	
Wisconsin	89.9 (83.7-96.1)	71.2 (62.5-79.9)		NR		75.6 (71.7-79.5)		67.0 (61.9-72.1)		65.5 (59.4-71.6)	
Wyoming	84.7 (77.9-91.5)	63.9 (54.8-73.0)		NR		69.8 (65.4-74.2)		63.6 (58.3-68.9)		51.5 (44.5-58.5)	
Median^c^	87.2	70.7		59.6		75.5		66.9		51.0	

**State**	**Unadjusted Prevalence Estimates**										

	**With Disability**						**Without Disability**				

	**Had a Medical Encounter %95% CI**		**Received Quit Advice% (95% CI)**		**Discussed Tx Options% (95% CI)**		**Had a Medical Encounter% (95% CI)**		**Received Quit Advice% (95% CI)**		**Discussed Tx Options% (95% CI)**

Arizona	93.4 (89.2-97.6)		NR		NR		78.3 (72.3-84.3)		57.7 (49.3-66.1)		NR
Arkansas	87.9 (81.9-93.9)		62.9 (55.6-70.2)		56.9 (47.0-66.8)		77.8 (74.0-81.6)		57.2 (52.0-62.4)		48.7 (41.8-55.6)
Colorado	86.5 (80.2-92.8)		83.3 (76.4-90.2)		NR		67.6 (62.7-72.5)		70.1 (64.6-75.6)		52.8 (45.9-59.7)
Delaware	92.6 (88.3-96.9)		68.1 (58.6-77.6)		NR		83.8 (79.8-87.8)		70.7 (65.1-76.3)		55.1 (48.1-62.1)
Iowa	88.3 (82.0-94.6)		71.4 (62.8-80.0)		NR		74.0 (70.1-77.9)		59.1 (54.3-63.9)		54.2 (48.0-60.4)
Kentucky	86.2 (81.7-90.7)		73.3 (67.0-79.6)		59.1 (51.5-66.7)		65.9 (61.0-70.8)		69.5 (64.0-75.0)		57.9 (50.7-65.1)
Louisiana	79.1 (73.7-84.5)		68.1 (60.4-75.8)		52.8 (44.8-60.8)		67.6 (64.5-70.7)		60.6 (56.7-64.5)		50.8 (45.4-56.2)
Montana	89.1 (84.2-94.0)		70.0 (62.7-77.3)		NR		75.0 (70.4-79.6)		63.8 (58.0-69.6)		49.4 (41.6-57.2)
Nebraska	86.1 (81.2-91.0)		69.3 (62.4-76.2)		62.5 (53.6-71.4)		71.9 (68.2-75.6)		60.1 (55.8-64.4)		46.9 (41.1-52.7)
New Jersey	89.0 (84.4-93.6)		75.2 (70.0-80.4)		56.3 (48.7-63.9)		76.7 (73.7-79.7)		68.1 (64.8-71.4)		49.8 (45.6-54.0)
New York	84.9 (78.9-90.9)		74.8 (67.5-82.1)		62.2 (54.0-70.4)		72.3 (68.3-76.3)		68.8 (64.3-73.3)		54.2 (48.8-59.6)
North Carolina	89.0 (86.2-91.8)		74.0 (69.7-78.3)		61.6 (56.2-67.0)		71.3 (68.6-74.0)		70.2 (67.2-73.2)		59.5 (55.9-63.1)
North Dakota	88.0 (80.7-95.3)		NR		NR		71.2 (66.1-76.3)		59.1 (52.7-65.5)		48.5 (40.3-56.7)
Ohio	83.6 (73.8-93.4)		71.4 (62.3-80.5)		NR		79.3 (74.4-84.2)		68.8 (62.2-75.4)		48.1 (39.3-56.9)
South Carolina	86.9 (81.1-92.7)		72.2 (66.4-78.0)		55.7 (47.9-63.5)		76.1 (72.8-79.4)		66.6 (62.4-70.8)		52.7 (47.5-57.9)
Texas	85.7 (80.6-90.8)		69.8 (62.5-77.1)		NR		64.1 (59.8-68.4)		57.7 (52.6-62.8)		43.1 (36.5-49.7)
Virginia	91.9 (87.7-96.1)		71.2 (63.3-79.1)		64.8 (56.1-73.5)		77.1 (72.8-81.4)		65.6 (60.5-70.7)		57.4 (51.1-63.7)
West Virginia	91.8 (88.4-95.2)		75.4 (69.4-81.4)		61.0 (52.7-69.3)		76.2 (71.8-80.6)		68.2 (63.1-73.3)		55.5 (48.8-62.2)
Wisconsin	89.2 (82.8-95.6)		71.3 (62.9-79.7)		NR		74.4 (70.4-78.4)		67.4 (62.4-72.4)		68.7 (62.9-74.5)
Wyoming	85.7 (79.3-92.1)		65.2 (56.8-73.6)		54.0 (44.0-64.0)		70.5 (66.3-74.7)		63.1 (57.9-68.3)		54.0 (47.1-60.9)
Median^c^	88.0		71.4		59.1		74.2		66.1		52.8

CI indicates confidence interval; Tx, treatment; NR, not reported.

a People aged ≥18 years.

b BRFSS does not report data if the sample size is fewer than 50 or the confidence interval is greater than 10. Consequently, prevalence estimates for some states and the U.S. Virgin Islands are not reported.

c If analysis is limited to states for which we are able to report estimates by disability status, age-adjusted median prevalences are 67.1% for people without disability who received advice to quit and 53.5% for people without disability who discussed treatment options with their provider.
